# A 5'-region polymorphism modulates promoter activity of the tumor suppressor gene MFSD2A

**DOI:** 10.1186/1476-4598-10-81

**Published:** 2011-07-07

**Authors:** Francesca Colombo, Felicia S Falvella, Antonella Galvan, Elisa Frullanti, Hideo Kunitoh, Toshikazu Ushijima, Tommaso A Dragani

**Affiliations:** 1 Department of Predictive and Preventive Medicine, Fondazione IRCCS Istituto Nazionale Tumori, Milan, Italy; 2 National Cancer Center Research Institute, Tokyo, Japan

## Abstract

**Background:**

The MFSD2A gene maps within a linkage disequilibrium block containing the MYCL1-*EcoRI *polymorphism associated with prognosis and survival in lung cancer patients. Survival discrepancies between Asians and Caucasians point to ethnic differences in allelic frequencies of the functional genetic variations.

**Results:**

Analysis of three single-nucleotide polymorphisms (SNPs) mapping in the MFSD2A 5'-regulatory region using a luciferase reporter system showed that SNP rs12072037, in linkage disequilibrium with the MYCL1-*EcoRI *polymorphism and polymorphic in Asians but not in Caucasians, modulated transcriptional activity of the MFSD2A promoter in cell lines expressing AHR and ARNT transcription factors, which potentially bind to the SNP site.

**Conclusion:**

SNP rs12072037 modulates MFSD2A promoter activity and thus might affect MFSD2A levels in normal lung and in lung tumors, representing a candidate ethnically specific genetic factor underlying the association between the MYCL1 locus and lung cancer patients' survival.

## Background

A 106-kb linkage disequilibrium (LD) block on chromosome 1p34, which includes the TRIT1, MYCL1, and MFSD2A genes, is associated with lung cancer prognosis and survival [1], although conflicting results of the association of this region with prognosis, in particular of the MYCL1-*Eco*RI polymorphism, have been reported [2,3]. Indeed, association of MYCL1-*EcoRI *with lung cancer patients' survival was observed in all of 4 studies of Asians, but in none of 3 studies on Caucasians [3]. The discrepancies might reflect ethnic differences in allelic frequencies of the functional genetic variants mapping in this locus, as suggested by the significant difference between Caucasian and Asian subjects in the frequencies of several SNPs located in the TRIT1, MYCL1, and MFSD2A gene [1].

Modulation of expression of a gene mapping in the MYCL1 region may represent a mechanism underlying the association of this region with cancer patients' survival. Indeed, MYCL1 expression is not detected in normal or tumor tissue. Both the TRIT1 and MFSD2A genes are downregulated in lung adenocarcinomas (ADCA), whereas overexpression of either gene has tumor-suppressor effects [1,4,5]. The MFSD2A gene was also strongly downregulated in a panel of non-small cell lung cancer (NSCLC) cell lines, where it inhibits cell adhesion and migration when overexpressed [5]. Thus, available data suggest that downregulation of MFSD2A plays a role in lung tumor progression.

Since functional polymorphisms in the promoter region may affect mRNA levels of target genes by altering transcription factor (TF) binding sites [6,7], we analyzed three single-nucleotide polymorphisms (SNPs) (rs3131703, rs12072037, and rs3738668) mapping in the MFSD2A 5' region for a potential role in altering MFSD2A promoter activity.

## Results

### SNP rs3131703 in the MFSD2A 5' regulatory region has no functional effects on transcriptional activity

Among the MFSD2A 5' region SNPs, only rs3131703, located 1284 bp upstream of the start codon, has detectable allele frequencies in Caucasians. Genotyping of this SNP in 151 Italian lung adenocarcinoma patients revealed a minor allele frequency (MAF) = 0.46, i.e., slightly higher than the allele frequency reported in the HapMap database for Caucasians (0.39, Table 1).

**Table 1:** Allele frequency of the three validated SNPs in MFSD2A 5' region by ethnicity

SNP	Chromosome position ^a^	Alleles ^b^	Frequency of the minor frequency allele (MAF) in HapMap populations ^a^	
			
			Caucasians (CEU)	Asians (JPT)
rs3131703	40192268	G/A	0.39	0.03
rs12072037	40192793	C/A	0.01	0.47
rs3738668	40193306	C/A	0.01	0.48

^a ^Chromosome 1 position and allele frequencies extracted from the HapMap data (http://www.hapmap.org), accessed on August 17, 2010. ^b ^Common/rare in Caucasians.

Analysis by quantitative real-time (qRT)-PCR to test for an association between genotype and MFSD2A mRNA levels revealed no significant difference between two genotype groups of normal lung tissue samples from 20 subjects in our series selected for homozygosity at either allele (10 GG versus 10 AA samples) (data not shown).

**Figure 1:**
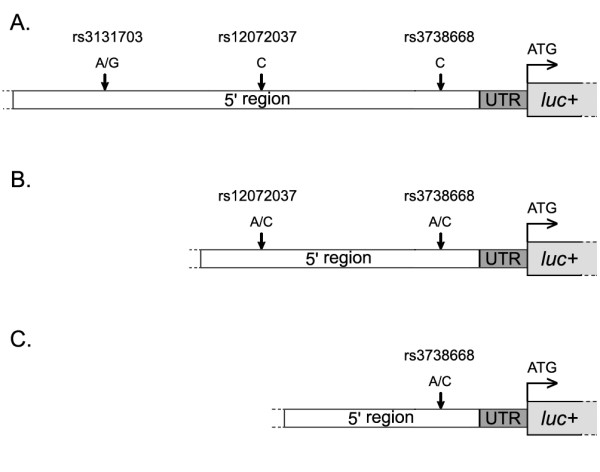
**Recombinant vectors used to assay MFSD2A promoter activity**. Schematic representation of recombinant vectors containing the firefly luciferase reporter gene under the control of three MFSD2A 5' region fragments of different length (panels A to C). Arrows indicate the position of the three polymorphisms investigated (see Materials and Methods for details; UTR, untranslated region; luc+, firefly luciferase gene).

To study the functional role of rs3131703, a 1499-bp fragment of the proximal promoter of the MFSD2A gene containing either of the two alleles (G/A) was subcloned into the pGL3-Basic vector upstream of the ATG of the firefly luciferase gene (Figure 1A) and transfected together with the *Renilla *luciferase pRL-TK reporter vector into different cell lines (A549, Hek293T, HepG2, HT-29, IGROV1, NCI-H520 and NCI-H596) for analysis by Dual-Luciferase Reporter Assay. While the MFSD2A promoter fragment containing SNP rs3131703 showed functional transcriptional activity, since the normalized firefly/Renilla luciferase activity was > 100-fold that of the empty vector in Hek293T cells, the two allelic variants of rs3131703 showed no statistically significant differences in promoter activity in any of 7 cell lines assayed, except for a weak effect of the G versus A allele in Hek293T (1.1-fold higher activity; P = 0.011, ANOVA) and NCI-H596 (1.1-fold increase, P = 0.007, ANOVA) cells.

### SNP rs12072037 is the major polymorphism involved in modulation of MFSD2A promoter activity

The MFSD2A 5' region rs12072037 (C/A alleles) and rs3738668 (C/A alleles) SNPs, mapping at -759 bp and -246 bp from ATG, respectively, are not polymorphic in Caucasians, whereas in Asian populations, the frequencies of the minor allele of these polymorphisms are quite high (MAF = 0.47-0.48; Table 1). Accordingly, sequencing analysis in a small number of Italian (n = 15) and Japanese (n = 15) lung cancer patients showed that SNPs rs12072037 and rs3738668 defined two haplotypes in Asians, AA and CC, whereas only the CC haplotype was detected in Caucasians.

**Figure 2:**
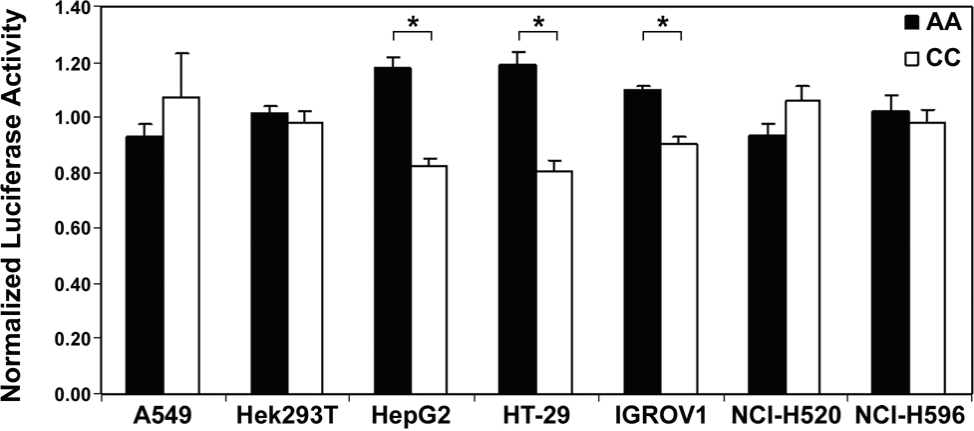
**MFSD2A promoter activity modulated by genetic polymorphisms**. Luciferase activity in cell lines transfected with recombinant plasmids containing the MFSD2A 5' region haplotypes (CC and AA) of SNPs rs12072037 and rs3738668, respectively. Data are mean ± S.E. of normalized luciferase activity.

To study the functional role of rs12072037 and rs3738668, we first tested the promoter activity of a 933-bp fragment of the MFSD2A gene proximal promoter containing the two linked SNPs (AA and CC haplotypes; Figure 1B) and subcloned into the pGL3-Basic vector upstream of the ATG of the firefly luciferase gene. Three of the 7 cancer cell lines transfected with this construct showed a significant difference between the two haplotypes in promoter activity. Indeed, HepG2, HT-29, and IGROV1 cells showed 1.4-, 1.4-, and 1.2-fold higher luciferase values, respectively, associated to the AA haplotype (present in Asians) as compared to the CC haplotype (the only one detected in our Italian samples) (P < 1.0 × 10^-6 ^for each cell line, ANOVA; Figure 2). Both haplotypes showed functional promoter activity, as indicated by the > 100-fold increase in luciferase activity in Hek293T cells.

Similar analyses using a 699-bp fragment containing only SNP rs3738668 (A and C alleles; Figure 1C) revealed no overall modulation of MFSD2A promoter activity, as indicated by luciferase values, except for a marginally statistically significant effect only in Hek293T cells (P = 0.022). As reported for the other constructs, a > 100-fold increase in luciferase activity in Hek293T cells was observed in the presence of either alleles of rs3738668 SNP.

These findings point to SNP rs12072037 as the main modulator of MFSD2A promoter activity.

### SNP rs12072037 alleles create different putative transcription factor binding sites

**Figure 3:**
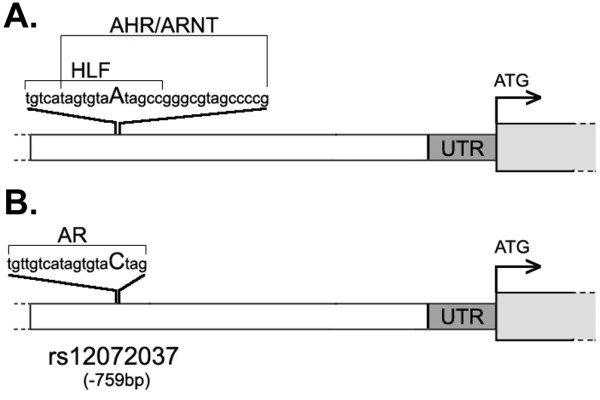
**In silico analysis of MFSD2A promoter binding sites**. Schematic representation of the putative TF binding sites created by the presence of the C (panel A) or A (panel B) allele of SNP rs12072037 in the MFSD2A 5' region, predicted by MatInspector Release professional 8.0. Sequences of the TF binding sites are shown, with alleles of rs12072037 shown in upper case. White boxes: 5' region; dark-grey boxes: 5'-UTR; light-grey boxes: coding region.

To determine whether SNP rs12072037 might modulate MFSD2A promoter activity by altering putative transcription factor (TF) binding sites in the MFSD2A 5' region, we first predicted the potential differential TF binding sites according to the presence of the A or C allele of the polymorphism using MatInspector Release professional 8.0 [8]. In the presence of the A allele, binding sites for HLF (hepatic leukemia factor) and for the AHR/ARNT (aryl hydrocarbon receptor/aryl hydrocarbon receptor nuclear translocator) heterodimer are generated (Figure 3A), whereas the same binding sites are lost in the presence of the C allele, which instead leads to a binding site for AR (androgen receptor) (Figure 3B).

**Figure 4:**
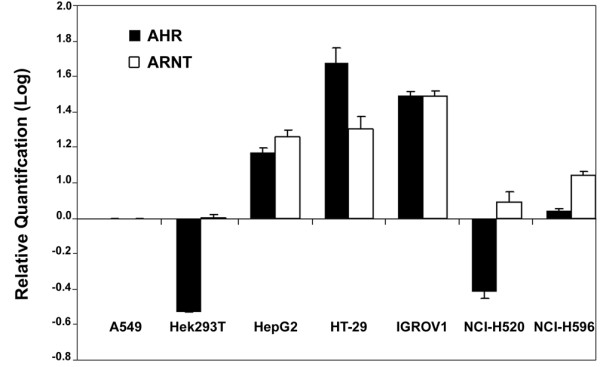
**Cell line-specific expression of transcriptional factors putatively binding to the MFSD2A promoter**. mRNA expression levels of AHR and ARNT in the 7 cell lines tested for luciferase activity. Data are mean ± S.E. of logarithms of RQ values.

qRT-PCR analysis of the four TFs potentially binding at the SNP rs12072037 site in the cell lines revealed high-level expression of both AHR and ARNT in HepG2, HT-29 and IGROV1 cells (Figure 4), in which higher MFSD2A promoter activity in the presence of the construct containing the A allele of rs12072037 was also observed, whereas mRNA expression levels of the two other TFs, i.e., AR and HLF in the different cell lines showed no apparent correlation with MFSD2A promoter activity (not shown).

To test the binding of AHR/ARNT heterodimer to the construct containing the A allele, a preliminary chromatin immunoprecipitation (ChIP) assay was carried out using chromatin from cells transfected with either of the two constructs. Real-time PCR analysis revealed an approximately 2-fold increase of immunoprecipitated DNA in the presence of the Asian allele (not shown).

## Discussion

We investigated the possible functional role of three SNPs, located in the MFSD2A 5'-regulatory region and showing population-specific allele frequencies, in modulating MFSD2A promoter activity. Luciferase reporter assay indicated no such modulation by SNP rs3131703, polymorphic only in the Caucasian population, and MFSD2A mRNA levels in normal lung tissue of lung cancer patients showed no statistically significant association with rs3131703, thus excluding the candidacy of this SNP as a functional genetic element regulating MFSD2A promoter activity. By contrast, analyses of SNPs rs12072037 and rs3738668, polymorphic in Asians, pointed to a functional role for rs12072037 in modulating MFSD2A transcriptional activity, showing allele-specific promoter activity in 3 of 7 cell lines.

Since promoter activity depends on the TF expression profile [9], a SNP in a regulatory region could modulate gene expression by altering TF binding sites, eliminating the natural site or generating a novel one and thus altering binding strength [10].

To investigate the basis of the observed cell type-specific differential effects on transcriptional activity, we measured expression of *in silico-*predicted TFs in the cell lines used for promoter activity analysis. Transcriptional levels of both AHR and ARNT were high in the same three cell lines (HepG2, HT-29, and IGROV1) showing higher MFSD2A promoter activity associated with the A allele of rs12072037.

Accordingly, AHR and ARNT specifically bind to the MFSD2A promoter containing the A allele of the rs12072037 polymorphism. Moreover, NCI-H520 and NCI-H596 cells, in which no modulation of MFSD2A promoter activity by genetic polymorphisms was detected, showed high levels of ARNT, but not of AHR, suggesting that the AHR/ARNT heterodimer rather than the single elements is necessary to increase MFSD2A promoter activity, as reported for other target genes [11]. Thus, the cell type-specific modulation of reporter mRNA levels by the MFSD2A promoter might be associated to the joint expression of AHR and ARNT, which may form a dimer upregulating the MFSD2A promoter variant containing the A allele at rs12072037. Our preliminary ChIP experiment supported this hypothesis since differences in immunoprecipitated DNA levels were measured between the A and C alleles of rs12072037. However, transfection of recombinant plasmids in cancer cells where the endogenous MFSD2A gene is strongly downregulated may not be adequate to test the TFs modulating the natural MFSD2A promoter in normal cells. To this aim, comparison of AHR and ARNT binding to the MFSD2A promoter between normal cells carrying the different rs12072037 genotypes would be required. Also, the MFSD2A promoter fragment that we have transfected contains an additional AHR/ARNT binding site downstream to the assayed site, causing a potential technical bias in the ChIP assay. Therefore, we cannot exclude a role for other TFs in affecting MFSD2A promoter activity.

SNP rs12072037, which modulated MFSD2A transcriptional activity, showed the most statistically significant differences of allele frequencies between Caucasian and Asian subjects among 12 SNPs mapping in the LD block [1]; indeed, SNP rs12072037 is almost invariant in Caucasians (Table 1). Furthermore, in Asians, the rs12072037 functional promoter polymorphism shows a significant LD with the MYCL1 SNP rs3134613 (*EcoRI*) (D' = 0.75, r^2 = 0.48, n = 88; genotypes of JPT samples downloaded from HapMap, accessed on August 24, 2009; analysis carried out using the JLIN program [12]), a SNP reported to be associated with prognostic factors and survival of lung cancer patients in Asian patients [3,13,14]. Together, these data suggest the candidacy of rs12072037 as the functional variation in the MYCL1 locus responsible for modulating nodal status, metastasis occurrence, and survival of lung cancer patients of Asian ethnicity.

## Conclusion

We identified SNP rs12072037 as the major polymorphism modulating MFSD2A promoter activity. Further studies in Asian lung cancer patients are needed to test the association of rs12072037 genotypes with MFSD2A mRNA levels in normal lung tissue and to clarify the role of this polymorphism in lung cancer progression and prognosis.

## Materials and Methods

### Cell lines

Human lung cancer (A549, NCI-H520, NCI-H596), hepatocellular carcinoma (HepG2), colorectal adenocarcinoma (HT-29), ovarian adenocarcinoma (IGROV1) and embryonal kidney (Hek293T) cells were used. All represent adherent epithelial cells. Each cell line was propagated in the appropriate culture medium as recommended by American Type Culture Collection, except for IGROV1 cells, which were cultured in RPMI 1640 medium with 2 mM L-glutamine, 1.5 g/l sodium bicarbonate, 10 mM HEPES, 1.0 mM sodium pyruvate, 4.5 g/l glucose and 10% FBS.

### Search and genotyping of MFSD2A SNPs in 5'-region

Genomic DNAs were obtained from lung ADCA patients (n = 151) enrolled at Istituto Nazionale Tumori, Milan, Italy, and from lung cancer cases (n = 15) at the National Cancer Center, Tokyo, Japan. Patients gave written permission to use their biological material for research purposes, and study protocols were approved by the Institute committees for ethics.

SNP rs3131703 was genotyped in 151 Caucasian samples using pyrosequencing analysis on a PSQ96MA system (Biotage AB, Uppsala, Sweden), according to the manufacturer's instructions using specific primers reported in Additional file 1.

To identify haplotypes defined by SNPs rs12072037 and rs3738668, genomic DNAs of 15 Caucasian and 15 Asian lung cancer samples were PCR-amplified for 1160 bp spanning from the 5'-region to an initial part of intron 1-2 of MFSD2A using primer pairs reported in Additional file 1. Sequences were determined using an automatic sequencer (Applied Biosystems, Foster City, CA) and aligned and compared using Genomatix Dialign software (http://www.genomatix.de).

### Quantitative real-time PCR (qRT-PCR)

Total RNAs were extracted with TRIZOL^® ^Plus RNA Purification Kit (Invitrogen, Carlsbad, CA) from cell lines and 20 normal lung tissue samples excised during thoracic surgery for lung cancer at Istituto Nazionale Tumori, Milan, Italy. Total RNAs were reverse-transcribed using a 1:1 mix of oligo(dT)18 and random hexamer primers, according to the protocol recommended for the Transcriptor First-Strand cDNA Synthesis Kit (Roche). MFSD2A gene expression levels in lung samples were measured by qRT-PCR as described [5]. Transcription factor mRNA levels in cell lines were assayed by qRT-PCR using primer pairs listed in Additional file 1. Amplification mixtures contained cDNA template diluted in RNase-free water, 12.5 μl 2 × Power SYBR^® ^Green PCR Master Mix (Applied Biosystems) and 0.3 μM specific PCR primers in a final volume of 25 μl. The human hypoxanthine phosphoribosyltransferase 1 (HPRT1) gene was used as housekeeping control. Reactions were run in duplicate on the 7900HT System (Applied Biosystems). Each experiment was repeated three times. Relative changes in mRNA levels were assessed using the comparative cycle threshold (Ct) method, and relative quantities were calculated using the A549 sample as calibrator.

### Subcloning of MFSD2A 5'-region allelic variants into luciferase reporter vector

Genomic DNAs of previously genotyped Caucasian individuals were selected for the two different alleles (G/A) of rs3131703 (Figure 1A) to be amplified for the 5' region (1499 bp from ATG). Genomic DNAs of previously genotyped Caucasian or Asian individuals were selected for the CC or AA haplotype (rs12072037 and rs3738668), respectively, to be amplified for 933 bp upstream of ATG (Figure 1B). Finally, the shorter fragments (699 bp from ATG), containing either of the two alleles (A/C) of rs3738668 (Figure 1C), were PCR-amplified from the previously subcloned longer fragments (Figure 1B). PCR amplifications were carried out using BIO-X-ACT Short DNA Polymerase kit (Bioline, London, UK) and primer pairs listed in Additional file 1. Fragments were cloned into pGL3-Basic vector (Promega, Madison, WI) upstream of the firefly luciferase reporter gene using Rapid DNA Dephos & Ligation Kit (Roche, Basel, Switzerland), according to the manufacturer's instructions.

### Luciferase reporter assays

Seven human cell lines derived from different tissues were co-transfected with pGL3-Basic recombinant vectors containing the different fragments of the MFSD2A 5' region and the pRL-TK plasmid containing the Renilla gene (Promega), using FuGene HD transfection reagent (Roche) according to the manufacturer's instructions. Firefly luciferase was used as primary reporter to monitor mRNA levels, whereas Renilla luciferase served as control reporter for normalization. Cells were assayed for luciferase activity 48 h after transfection using the Dual-Luciferase Reporter Assay System kit (Promega), according to the manufacturer's instructions. Normalized signals were expressed as relative luciferase units. Data from at least 6 replicas were evaluated.

### Chromatin immunoprecipitation (ChIP) assay

HT-29 cells (2 × 10^6 ^cells/immunoprecipitation) were transiently transfected with the pGL3-Basic recombinant vectors containing the intermediate length fragment (Figure 1B) of the MFSD2A 5' region and after 24 hours crosslinking with 1% formaldehyde was carried out. Cell lysis, micrococcal nuclease digestion of chromatin, immunoprecipitation (IP) and DNA recovery were carried out following the protocol of the Pierce Agarose ChIP kit (Pierce, Thermo Scientific, Rockford, IL). IP was carried out using anti-AHR and anti-ARNT transcription factors (MA1-513 and PA3-16508 respectively, Thermo Scientific) or normal rabbit IgG, as negative control. Recovered and purified DNA was quantified with Nanodrop Spectrophotometer ND-1000 (NanoDrop products, Wilmington, DE, USA) and quantitative real-time PCR was carried out with primers flanking the transcription factor binding site (Additional File 1). DNA levels were calculated by subtracting the Ct of the negative control sample from the Ct of the immunoprecipitated sample, to correct for the plasmidic DNA carry-over.

### Statistical analyses

Associations of quantitative measurements with genotypes or MFSD2A promoter variants were assessed by analysis of variance, using the Rcmdr package in R [15].

## Competing interests

This work was funded in part by grants from Associazione and Fondazione Italiana Ricerca Cancro (AIRC and FIRC), Fondo Investimenti Ricerca di Base (FIRB), Italy. The authors declare no conflict of interest.

## Authors' contributions

All authors read and approved the final manuscript. FC and FSF contributed to the design of the study, *in vitro *functional assays, and in drafting the manuscript; FSF, AG, and EF contributed to the molecular studies; HK and TU contributed to the Asian DNA samples; TAD contributed to data analysis and drafting of the manuscript.

## Supplementary Material

Additional file 1 **List of primers used**. Click here for file
