# Supplementary material for: A 5'-region polymorphism modulates promoter activity of the tumor suppressor gene MFSD2A
Source: Mol Cancer. 2011 Jul 7;10:81. doi: 10.1186/1476-4598-10-81 (PMC3155907; doi:10.1186/1476-4598-10-81)
Supplement: Additional file 1 — List of primers used. [file 1476-4598-10-81-S1.DOC]

| Additional file 1. PCR primers used for amplification, genotyping and subcloning of MFSD2A 5’ region or for qRT-PCR to measure mRNA expression levels of TFs binding to SNP rs12072037, or immunoprecipitated DNA after ChIP assay. | | | | |
| --- | --- | --- | --- | --- |
| Forward primer | Reverse primer | Reference sequence | Gene target | Use |
| 5’-aggcctggctctgctacata-3’ | 5’-ctctctggctcgtaggcttc-3’ | NT_032977 | MFSD2A,  5’ region | Amplification of 1160 bp containing SNPs rs12072037 and rs3738668 |
| 5’-gtctttagttccacctctgagtcc-3’ | 5’-cttatctctcctccctgcaatca-3’ | rs3131703 | MFSD2A,  5’ region | Amplification for genotyping SNP rs3131703 |
| 5’-tggaactctcaaaggc-3’ |  | rs3131703 | MFSD2A,  5’ region | Sequence primer for genotyping SNP rs3131703 by pyrosequencing |
| 5’-ctattataccatgggccattccctttccacagta-3’ | 5’-ctattataccatggcccgcgggccacgccgct-3’ | NT_032977 | MFSD2A,  5’ region | Amplification and subcloning of a 1499-bp fragment containing SNP rs3131703 into pGL3-Basic vector |
| 5’- ctattataccatggaggcctggctctgctacata-3’ | 5’-ctattataccatggcccgcgggccacgccgct-3’ | NT_032977 | MFSD2A,  5’ region | Amplification and subcloning of a 933-bp fragment containing SNPs rs12072037 and rs3738668 into pGL3-Basic vector |
| 5’-ctattataccatggccactggattgcctcatctt-3’ | 5’-ctattataccatggcccgcgggccacgccgct-3’ | NT_032977 | MFSD2A,  5’ region | Amplification and subcloning of a 699-bp fragment containing SNP rs3738668 into pGL3-Basic vector |
| 5’-gactttgctttccttggtcagg-3’ | 5’-tccttttcaccagcaagcttg-3’ | NM_000194 | HPRT | Housekeeping control for qRT-PCR |
| 5’-caaatcaccccccaggaatt-3’ | 5’-tcagcccatccactggaataat-3’ | NM_000044 | AR | qRT-PCR to assess mRNA expression levels of AR TF |
| 5’-tcccatctccgaactgtatgc-3’ | 5’-gcaacagctgacctggtctg-3’ | NM_002126 | HLF | qRT-PCR to assess mRNA expression levels of HLF TF |
| 5’-cgagtcccatatccgaatgatt-3’ | 5’-aaaactatcatgccactttctccag-3’ | NM_001621 | AHR | qRT-PCR to assess mRNA expression levels of AHR TF |
| 5’-tgtgaataggctgagctttgtga-3’ | 5’-gagccaagtccattcctgca-3’ | NM_001668  NM_178426  NM_178427 | ARNT (transcript variants 1, 2 and 3) | qRT-PCR to assess mRNA expression levels of ARNT TF |
| 5’-gtggtttttgagggccgaaagaga-3’ | 5’-gagagaagccggggctacgc-3’ | NT_032977 | MFSD2A,  5’ region | qRT-PCR to asses DNA levels of the AHR/ARNT binding site after ChIP assay |
| Primers used in subcloning the MFSD2A 5’-region have an overhanging 5’-tail, not complementary to the reference sequence and allowing *NcoI* restriction. Note that G in position 14 was modified from the original A to create the restriction site. | | | | |
